# A new species of hermit crab, *Diogenes heteropsammicola* (Crustacea, Decapoda, Anomura, Diogenidae), replaces a mutualistic sipunculan in a walking coral symbiosis

**DOI:** 10.1371/journal.pone.0184311

**Published:** 2017-09-20

**Authors:** Momoko Igawa, Makoto Kato

**Affiliations:** Graduate School of Human and Environmental Studies, Kyoto University, Kyoto, Japan; Evergreen State College, UNITED STATES

## Abstract

Symbiont shift is rare in obligate mutualisms because both the partners are reciprocally dependent on and specialized to each other. In the obligate accommodation–transportation mutualism between walking corals and sipunculans, however, an unusual saltatory symbiont shift was discovered. In shallow waters of southern Japan, an undescribed hermit crab species was found living in corallums of solitary scleractinian corals of the genera *Heterocyathus* and *Heteropsammia*, replacing the usual sipunculan symbiont. We described the hermit crab as a new species *Diogenes heteropsammicola* (Decapoda, Anomura, Diogenidae), and explored its association with the walking corals. This hermit crab species obligately inhabits the coiled cavity of the corals, and was easily distinguished from other congeneric species by the exceedingly slender chelipeds and ambulatory legs, and the symmetrical telson. Observations of behavior in aquaria showed that the new hermit crab, like the sipunculan, carries the host coral and prevents the coral from being buried. This is an interesting case in which an organism phylogenetically distant from Sipuncula takes over the symbiotic role in association with a walking coral. The hermit crab species is unique in that its lodging is a living solitary coral that grows with the hermit crab in an accommodation–transportation mutualism.

## Introduction

Obligate mutualism is a climax of co-evolution between symbiotic organisms. Because both partners of an obligate mutualism are reciprocally dependent on and specialized to each other, symbiont shift only rarely occurs. However, rare symbiont shifts often result in reciprocal diversification of the partners, especially in obligate pollination mutualisms between fig and fig wasp [1–2] and between leafflower and leafflower moth [3–5]. In these cases, host shifts have occurred only between phylogenetically related partners, and saltatory host shift between phylogenetically distant taxa is not known. We report here a novel saltatory host shift of partners in an obligate mutualism.

In marine ecosystems, the symbiosis between walking corals and sipunculans is a well-known example of obligate mutualism, where the partners respectively offer accommodation and transportation. Solitary scleractinian corals of the genera *Heterocyathus* and *Heteropsammia* inhabit marine soft bottoms without attaching to hard substrata. Their corallums each contain a coiled cavity inhabited by a sipunculid worm [6–10]. Observations of internal corallum structure suggest that a larval coral initially settles on a small gastropod shell that has already been colonized by a sipunculan, and that the coral grows over and ultimately beyond the shell, providing a coiled cavity for the equally growing worm partner. The coral serves as a sturdy shelter, protecting the worm against possible predators. Furthermore, the worm can roam the seafloor, carrying the host coral with it [11–14]. If the coral is overturned by water currents or buried by sedimentation, the worm assists the coral in recovering its upright position on the sea floor. Thus, the association between coral and sipunculan has been described as mutualistic [15–16]. However, the symbiotic sipunculan may be replaced by a hermit crab [10, 17–18]. This observation raises the intriguing question whether the hermit crab is an alternative symbiont in the coral–sipunculan association.

During our survey of benthic fauna in Oshima Strait, north of Kakeroma Island, Ryukyu Islands, southern Japan, we found that some solitary scleractinian corals of the genera *Heterocyathus* and *Heteropsammia* were inhabited by hermit crabs, rather than by sipunculans. Morphological observations revealed that the hermit crab is a new species of the genus *Diogenes* Dana, 1851 [19], although all other known species of the genus are known to inhabit only molluscan shells.

The genus *Diogenes* belongs to the family Diogenidae and is typically characterized by the possession of an intercalary rostriform process flanked by ocular acicles, though several species exhibit a tendency for reduction of this process, e.g., “*Troglopagurus* group” [20]. Currently, 66 species are recognized worldwide, 63 of which are found in the Indo-West Pacific region [21–27]. Our morphological observations suggest that the corallum-inhabiting hermit crab belongs to the *D*. *edwardsii* species group as defined by Asakura and Tachikawa [28], which is characterized by an intercalary rostriform process unarmed on the lateral margins, an antennal peduncle distinctly longer than the ocular peduncle, and an antennal flagellum with a pair of long setae on the distal margin of each article ventrally. At present, the *D*. *edwardsii* species group includes 29 species from the Indo-West Pacific [22–29].

We carried out morphological, ecological, and behavioral observations of the unusual corallum-inhabiting hermit crab. In this paper, we describe and illustrate the hermit crab as a new species. In addition, we tested the hypothesis that the association between the hermit crab and the solitary coral is the same type of accommodation–transportation symbiosis observed between sipunculan and the coral through behavioral observations in aquaria. The study has shed light on how the saltatory symbiont shift from the sipunculan to the hermit crab has occurred.

## Materials and methods

### Sampling and observation

In our preliminary survey of walking corals in shallow waters around Kochi Prefecture, and the Amami and Okinawa Islands in southern Japan, only walking corals collected from the Amami Islands were sometimes inhabited by hermit crabs instead of sipunculans. Thus, we carried out extensive sampling of the walking corals with their symbiotic sipunculans and hermit crabs in Oshima Strait, Amami-Oshima Island. Permits for the research were obtained from the Forestry and Fisheries Promotion Division, Oshima Branch Office, Kagoshima Prefecture. Corals of the genera *Heterocyathus* and *Heteropsammia* are not endangered or protected. We dredged soft bottoms (28°12'N, 129°14'E, 40–80 m deep) using a small dredge (RIGO, Tokyo, Japan; mouth size = 40 × 15 cm, mesh size = 2 mm). We collected 3 *Hc*. *alternatus* and 19 *Hp*. *cochlea* individuals between 2012 and 2016; some of which were inhabited by hermit crabs. For observation on behavior, some hermit crab-inhabited corals were reared in aquaria with substrata collected by dredging. All specimens of the new hermit crab species were found in walking corals. All specimens were ultimately preserved in 99% ethanol.

Material examined in this study is deposited in the National Museum of Nature and Science (NSMT), Tokyo, Japan, and the Kyoto University Museum (KUZ). General hermit crab terminology follows McLaughlin *et al*. [30]. Shield length (sl), measured from the tip of the rostrum to the midpoint of the posterior margin of the shield, is used as an indicator of specimen size.

### Nomenclatural acts

The electronic edition of this article conforms to the requirements of the amended International Code of Zoological Nomenclature (ICZN), and hence the new species name contained herein is available under that Code from the electronic edition of this article. This published work and the nomenclatural acts it contains have been registered in ZooBank, the online registration system for the ICZN. The ZooBank LSIDs (Life Science Identifiers) can be resolved and the associated information viewed through any standard web browser by appending the LSID to the prefix “http://zoobank.org/”. The LSID for this publication is: urn:lsid:zoobank.org:pub: 2115C9F7-4521-4966-A226-07A1B456713B. The electronic edition of this work was published in a journal with an ISSN, and has been archived and is available from the following digital repositories: PubMed Central, LOCKSS.

## Results

### Taxonomic account

**Genus *Diogenes* Dana**, **1851**

**Fig 1:**
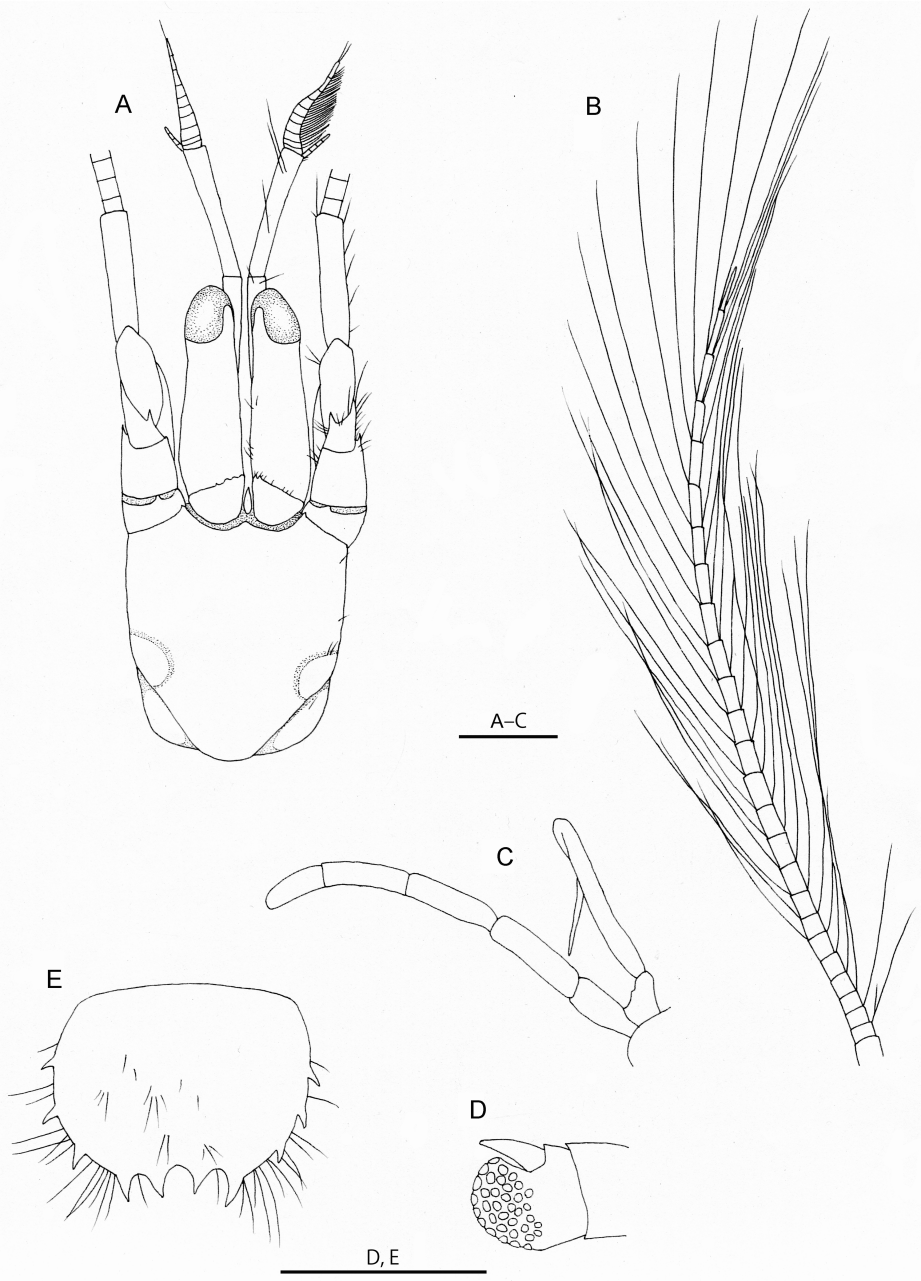
*Diogenes heteropsammicola* sp. nov. A, paratype, male (sl. 1.3 mm), NSMT-Cr 25561; B–E, holotype, male (sl. 1.3 mm), NSMT-Cr 25560. A, shield and cephalic appendages, dorsal view (setae on left side omitted); B, left antennal flagellum, dorsal view; C, left third maxilliped, lateral view (setae omitted); D, dactylus, propodus, and distal part of carpus of left fourth pereopod, lateral view (setae omitted); E, telson, dorsal view. Scale bars: 0.5 mm.

**Fig 2:**
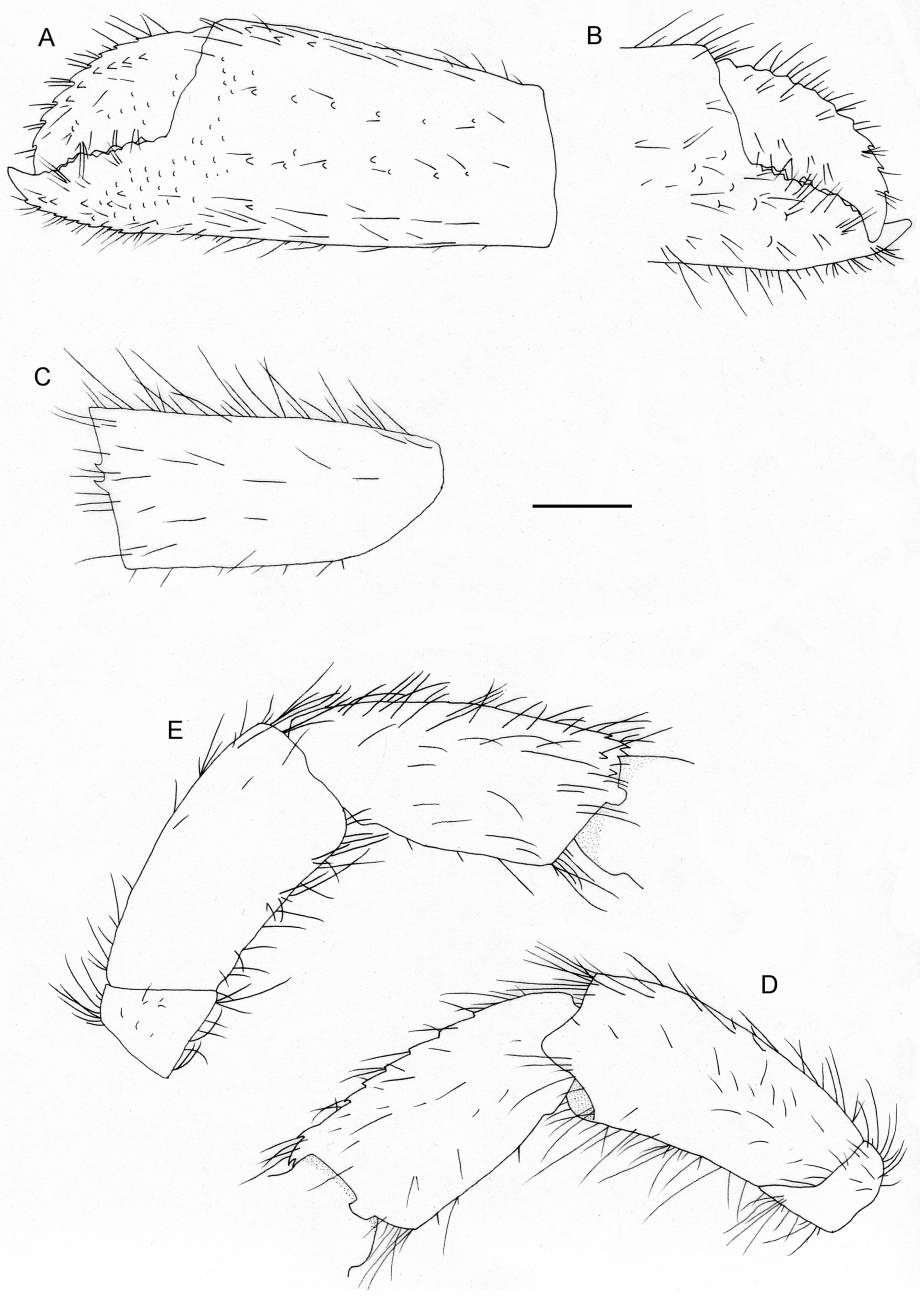
*Diogenes heteropsammicola* sp. nov., holotype, male (sl 1.3 mm), NSMT-Cr 25560. Left cheliped. A, chela, dorsal view; B, dactylus and fixed finger, ventral view; C, carpus, dorsal view; D, carpus and merus, lateral view; E, same, mesial view. Scale bar: 0.5 mm.

**Fig 3:**
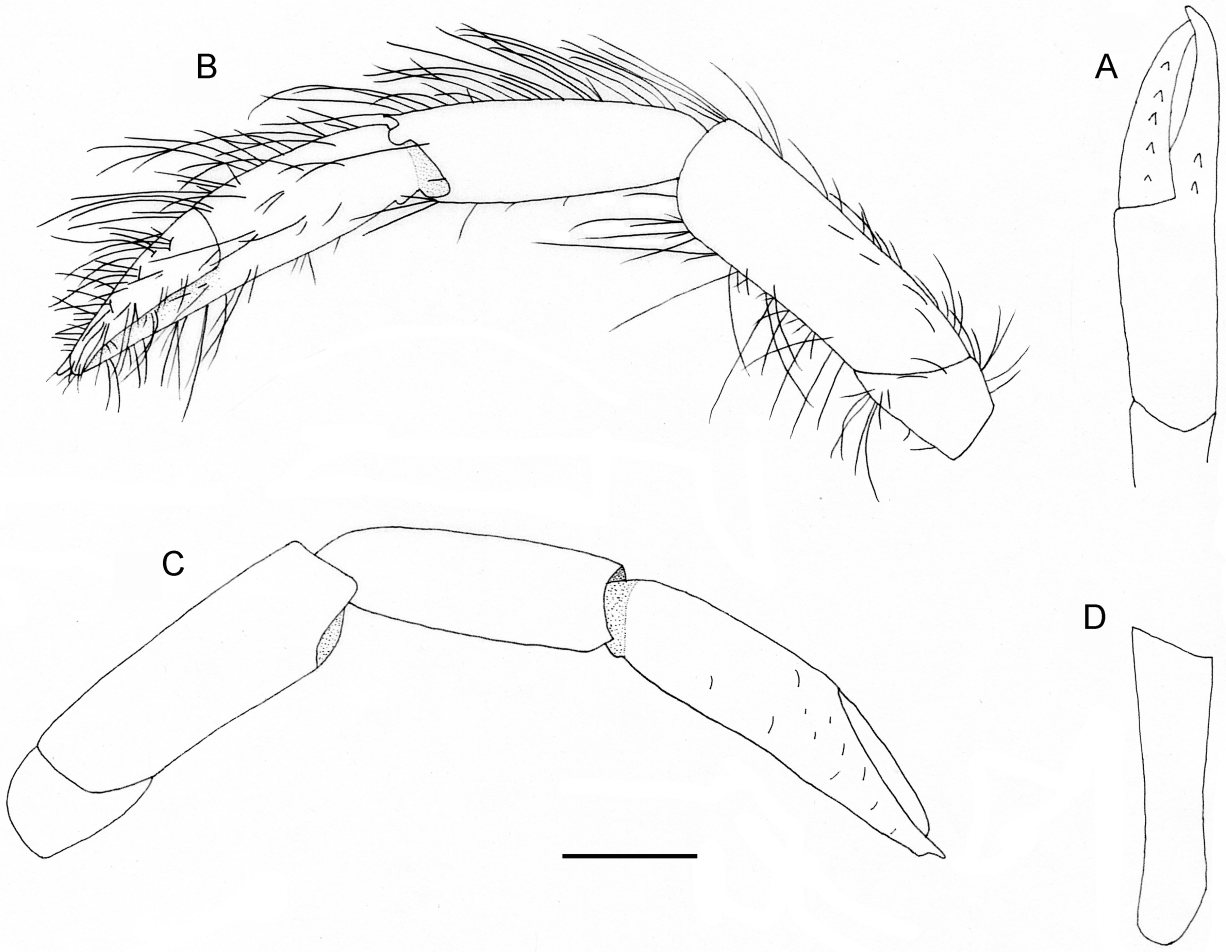
*Diogenes heteropsammicola* sp. nov., holotype, male (sl 1.3 mm), NSMT-Cr 25560. Right cheliped. A, chela, dorsal view (setae omitted); B, entire, mesial view; C, same, lateral view (setae omitted); D, carpus, dorsal view (setae omitted). Scale bar: 0.5 mm.

**Fig 4:**
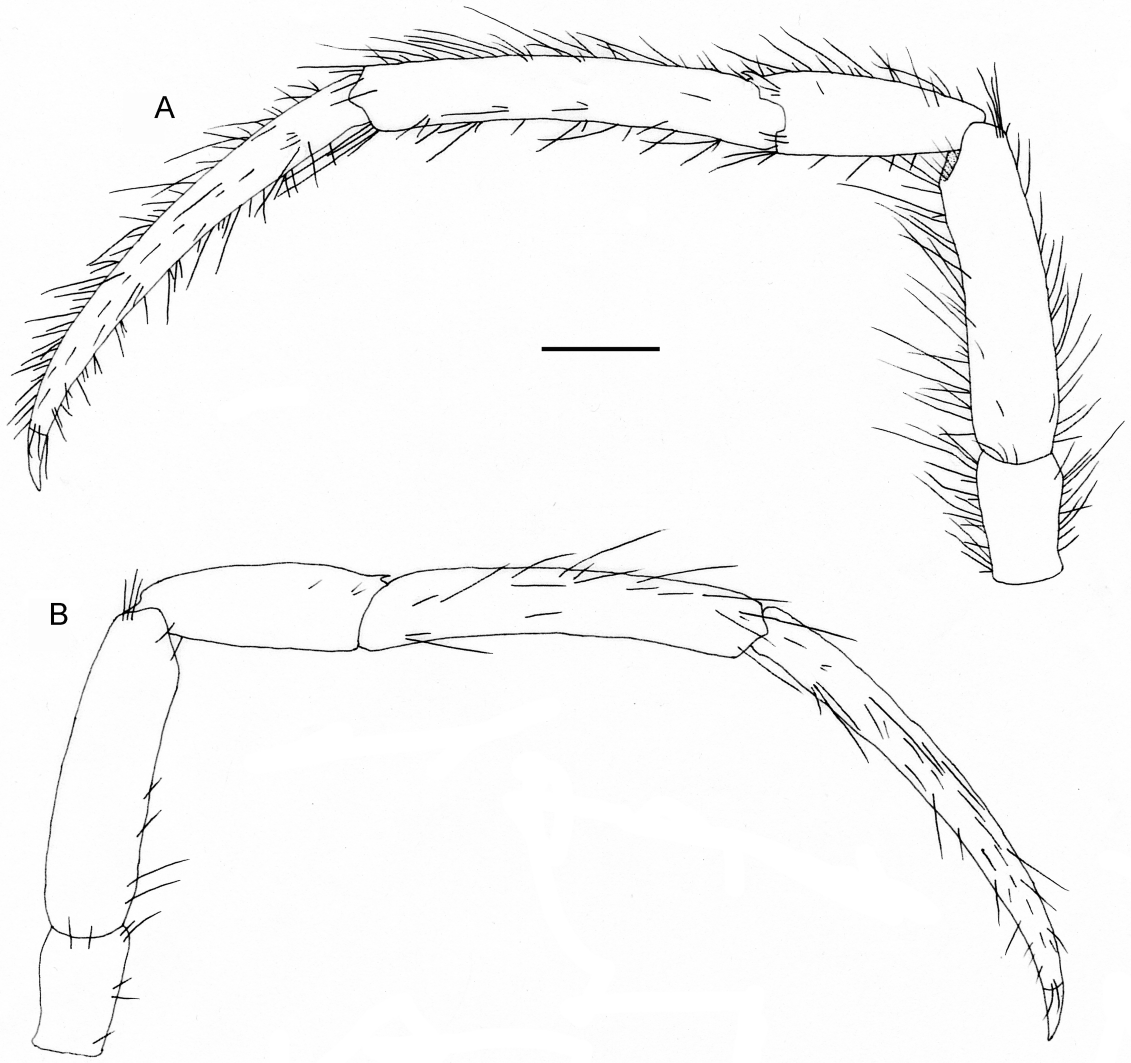
*Diogenes heteropsammicola* sp. nov., holotype, male (sl 1.3 mm), NSMT-Cr 25560. Left second pereopod. A, lateral view; B, mesial view (only setae on surface shown). Scale bar: 0.5 mm.

**Fig 5:**
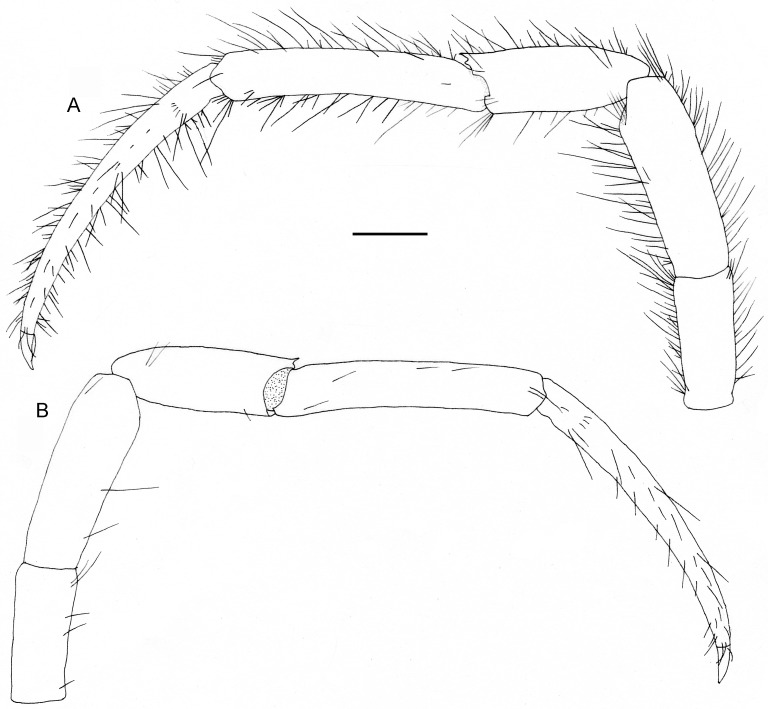
*Diogenes heteropsammicola* sp. nov., holotype, male (sl 1.3 mm), NSMT-Cr 25560. Left third pereopod. A, lateral view; B, mesial view (only setae on surface shown). Scale bar: 0.5 mm.

**Fig 6:**
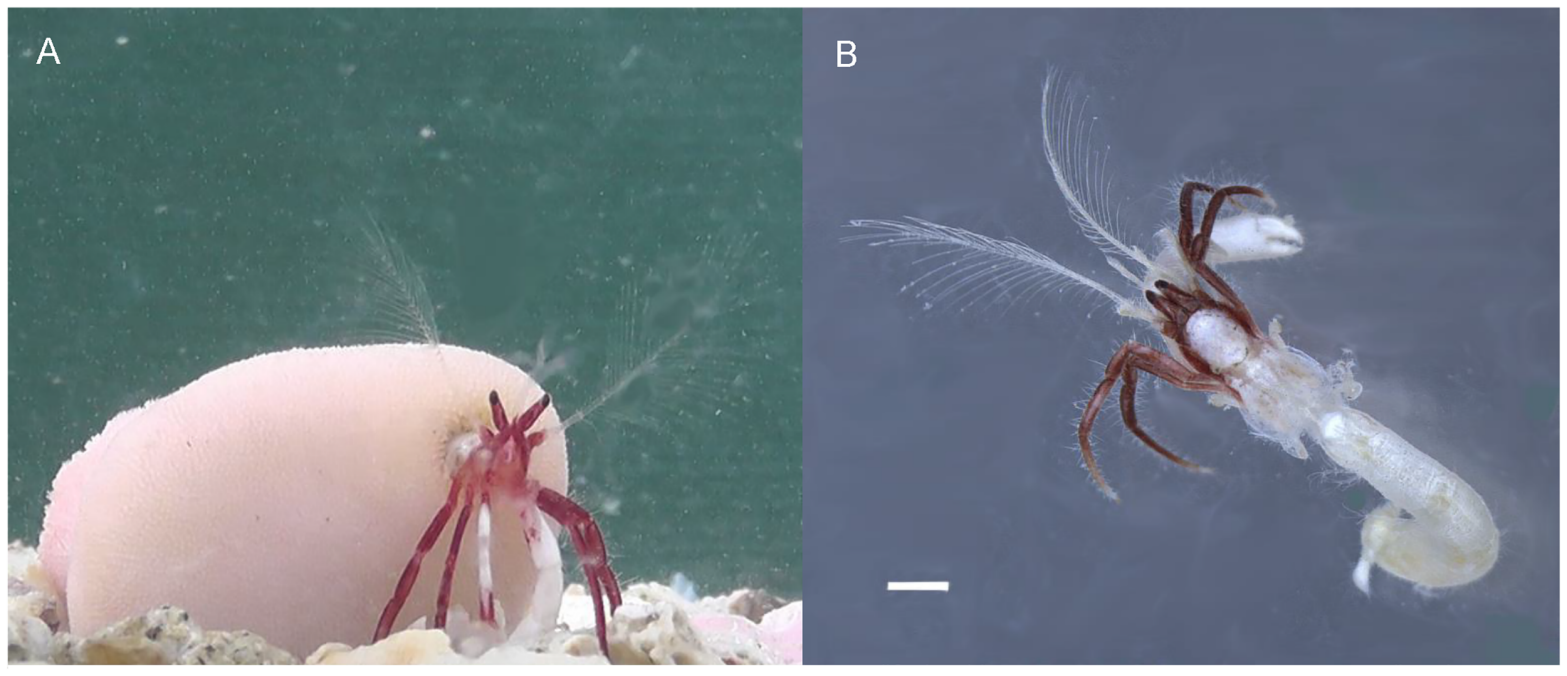
*Diogenes heteropsammicola* sp. nov. in life. A, an individual in an aquarium, carrying the coral; B, an individual removed from its host coral. Scale bar: 1 mm.

**Fig 7:**
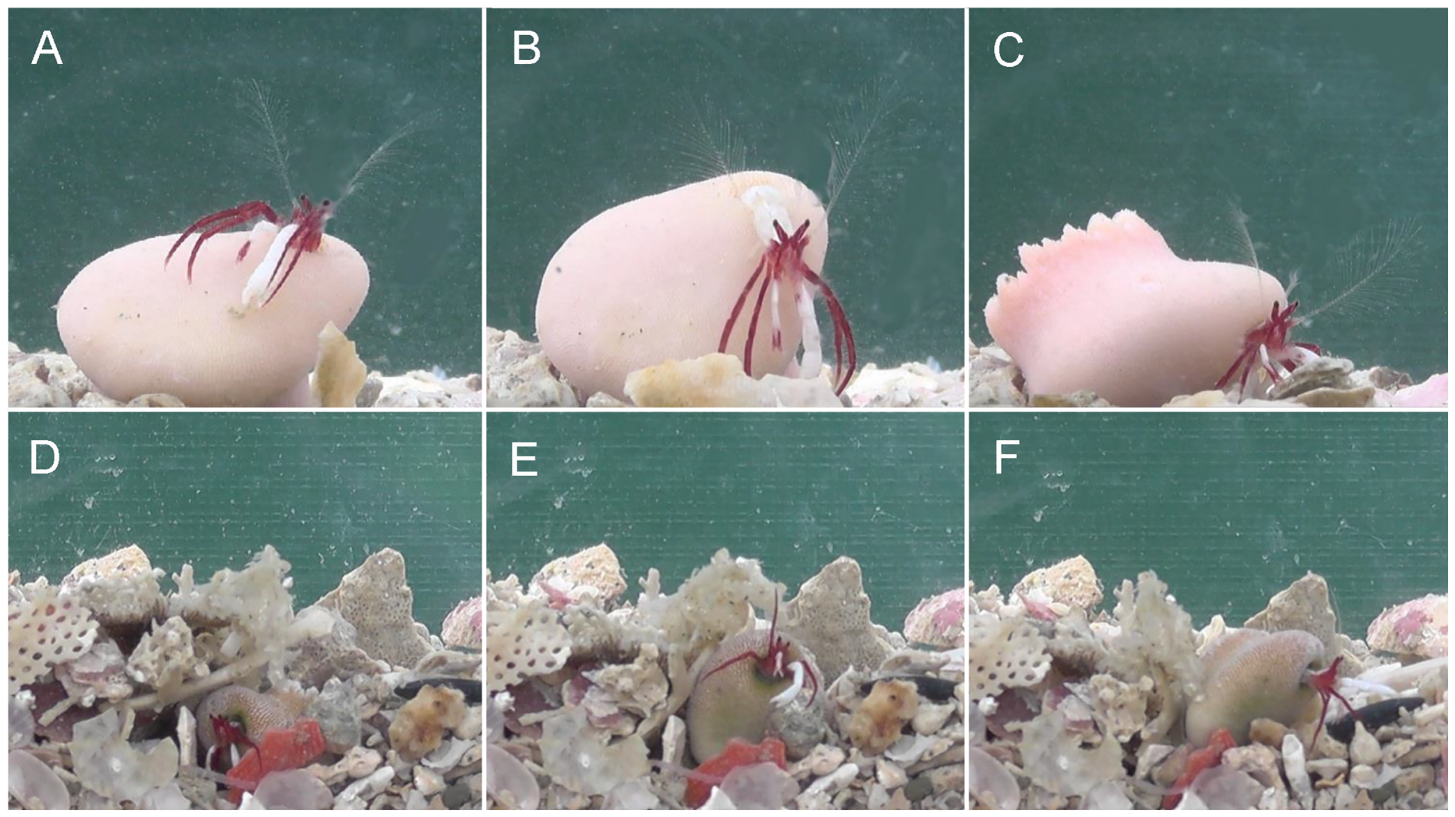
Behavior of *Diogenes heteropsammicola* sp. nov. A–C, sequence of behaviors to recover from an overturned to upright position in which the hermit crab leans out of the overturned coral (A), grasps the bottom with its ambulatory legs and left cheliped (B), and turns the coral upright using the pleon (C); D–F, sequence of behaviors to overcome burial in sediment, whereby the buried hermit crab (D) pushes away the sediment using its chelipeds and ambulatory legs (E), and then crawls away (F).

***Diogenes heteropsammicola* sp. nov.** urn:lsid:zoobank.org:act: 0D270CD7-EF12-4628-B4B3-DDD007905116 (Figs 1–7)

#### Material examined

Holotype: male (sl 1.3 mm), Oshima Strait, northeast of Kakeroma Island, Kagoshima, Japan 28°12.24'N, 129°14.48'E to 28°11.98'N, 129°14.78'E, 58–74 m, symbiotic with the coral *Heteropsammia cochlea*, 17 May 2015, NSMT-Cr 25560.

Paratypes: 1 male (sl 1.3 mm), Oshima Strait, northeast of Kakeroma Island, Kagoshima, Japan 28°12.37'N, 129°14.65'E to 28°12.22'N, 129°14.43'E, 65–75 m, symbiotic with the coral *Heteropsammia cochlea*, 16 November 2013, NSMT-Cr 25561; 1 male (sl 1.0 mm), Oshima Strait, northeast of Kakeroma Island, Kagoshima, Japan 28°12.43'N, 129°14.43'E to 28°12.18'N, 129°14.57'E, 58–74 m, symbiotic with the coral *Heterocyathus alternatus*, 14 May 2014, KUZ-Z1870; 1 female (sl 1.2 mm), Oshima Strait, northeast of Kakeroma Island, Kagoshima, Japan 28°12.23'N, 129°14.48'E to 28°12.44'N, 129°14.23'E, 62–77 m, symbiotic with the coral *Heteropsammia cochlea*, 29 November 2015, NSMT-Cr 25562.

Non-type: 1 female (sl 1.1 mm), Oshima Strait, northeast of Kakeroma Island, Kagoshima, Japan 28°12.2'N, 129°14.57'E to 28°12.13'N, 129°14.5'E, 40–50 m, symbiotic with the coral *Heteropsammia cochlea*, ovigerous, 16 November 2013.

#### Description

Shield (Fig 1A) slightly longer than broad, anterior margin between rostrum and lateral projections concave, anterolateral margins sloping, posterior margin roundly truncate, dorsal surface with few short setae. Rostrum short, broad, obtusely triangular, not produced beyond lateral projections; lateral projections triangular, acutely pointed.

Ocular peduncles (including corneas) (Fig 1A) about 0.8 times as long as shield, moderately stout, slightly inflated basally; corneas not dilated, corneal width about 0.3 of length of ocular peduncle; ocular acicles each with straight mesial margin, anterolateral margin nearly straight or slightly convex, bearing 3 or 4 minute or small spines decreasing in size laterally and not extending to entire length of lateral margin. Intercalary rostriform process not reaching anterior apexes of ocular acicles, slightly broadened medially, tapering acutely.

Antennular peduncles (Fig 1A) overreaching distal corneal margins by entire length of ultimate segment. Ultimate segment 4.0 times longer than distal width, subequal in length to penultimate segment, with some long setae on dorsal surface. Ultimate, penultimate and basal segments unarmed.

Antennal peduncles (Fig 1A) overreaching distal corneal margins by 0.5–0.6 length of fifth segment. Fifth segment with row of moderately short stiff setae on ventral surface. Fourth and third segments unarmed. Second segment with strong spine at dorsolateral distal angle. First segment unarmed. Antennal acicles short, each with a single spine slightly proximal to median part of mesial margin, terminating in slender spine. Antennal flagellum (Fig 1B) about 3.0 times longer than shield, articles with paired long setae.

Third maxilliped (Fig 1C) slender. Carpus unarmed on dorsodistal margin. Ischium-basis fused segment with 4 spinules on mesial surface, without crista dentata.

Left cheliped (Fig 2) elongate and slender; moderately setose (setae on merus and ischium plumose, those on carpus to chela simple). Chela 2.3–2.6 times longer than maximum width. Dactylus about half length of palm, curved distally and overlapped by fixed finger; mesial margin with double row of spines; dorsal surface with scattered tubercles; cutting edge sinuous, with row of blunt calcareous teeth. Palm subequal in length to carpus; dorsal surface with small tubercles and with double row of blunt spines along dorsomidline, dorsomesial margin with row of small spines. Fixed finger with scattered, small tubercles or spines on dorsal surface, dorsolateral margin with row of small spines; ventral surface with scattered tubercles proximally; cutting edge sinuous, with row of blunt calcareous teeth. Carpus 1.8–2.4 times longer than maximum width, with single row of spines increasing in size distally on dorsal margin; dorsodistal margin with 2 spines or unarmed (smaller individual); mesial surface with few spinules or unarmed. Merus distinctly longer than high, dorsal surface rounded. Ischium unarmed.

Right cheliped (Fig 3) slender, setose (setae on merus and ischium plumose, those on carpus to chela simple). Chela 3.5–5 times longer than wide; fingers crossing distally, with broad hiatus. Dactylus gently arched, about 0.6–0.9 times longer than palm; dorsal surface with single row of small spines along dorsomidline; mesial surface with several short setose ridges; ventral surface unarmed. Palm about 0.7–0.8 times longer than carpus; lateral and ventral surface with several short, setose ridges. Fixed finger gently curved, with some small spines on dorsal surface proximally or unarmed. Carpus with small spine on dorsodistal margin. Merus and ischium unarmed.

Ambulatory legs (Figs 4 and 5) long, slender, with moderately dense, long setae on dorsal and ventral margins (setae on carpi, meri, and ischia plumose, those on propodi to dactyli simple); third pair slightly longer than second pair. Dactyli 1.2–1.3 times longer than propodi and 10.5–12.0 times longer than high, each terminating in sharp, corneous claw; ventromesial margin unarmed. Propodi nearly straight or faintly ventrally curved, 1.6–1.8 times longer than carpi and about 6.0 times longer than high. Carpi 0.7–0.9 length of meri, each with small dorsodistal spine on dorsal margin. Meri and ischia unarmed.

Fourth pereopods chelate (Fig 1D). Dactyli reaching distal margins of propodi. Propodi each with dorsodistal margin not particularly produced, unarmed; propodal rasp well developed. Carpi unarmed.

Pleon curved. Male with unpaired, uniramous, left second to fifth pleopods. Female with unpaired, left second to fifth pleopods, second to fourth unequally biramous, fifth uniramous.

Uropods symmetrical, endopodal and exopodal rasps well developed.

Telson (Fig 1E) symmetrical, terminal margin with 2 spines; left and right lateral margin each with 2–3 spines; median cleft obsolete.

#### Coloration in life

Shield generally white. Ocular peduncles maroon red. Antennular peduncles with ultimate and penultimate segments translucent, basal segment maroon red. Antennal peduncles with first to third segments maroon red, fourth and fifth segments translucent. Chelipeds generally white; merus of left cheliped with tinge of reddish brown on mesial face; dactylus, palm, and fixed finger of right cheliped with tinge of reddish brown or maroon red on dorsal faces. Ambulatory legs generally maroon red, dactyli distally white.

#### Distribution

At present, known only from Oshima Strait, between Kakeroma Island and Amami-Oshima Island, Kagoshima, Japan, depths of 60–80 m, and Ikomo Bay, western coast of Kakeroma Island, depth of 31 m [18].

#### Remarks

*Diogenes heteropsammicola* sp. nov. belongs to the *D*. *edwardsii* species group because of the intercalary rostriform process being smooth on the lateral margins, the antennal peduncle distinctly overreaching the distal corneal margin, and the antennal flagellum bearing a pair of long setae on the distal margin of each article ventrally [28]. The new species is readily distinguished from all other species in this group by its exceedingly slender chelipeds and ambulatory legs, its symmetrical telson, red and white coloration, and the unique symbiotic habit with solitary corals.

#### Etymology

The new species is named after its mutualistic relationship with the solitary scleractinian corals of the genera *Heteropsammia*, keeping in mind that this hermit crab is also associated with *Heterocyathus* corals.

### Ecological and behavioral account

*Diogenes heteropsammicola* sp. nov. was obtained from shallow waters (depth of ca. 60–80 m) in Oshima Strait, where the periodic tidal current is strong even near the bottom. The bottom sediment was shelly sand, including numerous fragments of bryozoans, foraminiferans, and molluscan shells. The sand was also inhabited by lancelets.

The hermit crab was found only in the coiled cavity within solitary scleractinian corals of the genera *Heterocyathus* and *Heteropsammia*, and was not found in empty and naked gastropod shells. Observations in aquaria demonstrated that the hermit crab was ambulatory while carrying the host coral (Fig 6A). When the coral was overturned, the hermit crab leaned out of the corallum cavity to grasp the bottom with its long ambulatory legs and left cheliped, and then turned the coral to an upright position using the pleon (Fig 7A–7C). When the coral was buried, the hermit crab pushed away the sediment using its chelipeds and ambulatory legs, and then crawled away while still in the coral (Fig 7D–7F).

When feeding, the hermit crab filtered organic particles using the antennal flagella and third maxillipeds. The hermit crab worked the antennae in a circular motion, in which the right antenna made clockwise turns and the left antenna made counterclockwise turns. As a result, the hermit crab appeared to create an upward current near the third maxillipeds. If organic particles attached to the antennal flagellum, the hermit crab swept it clean using the third maxillipeds.

Some female specimens carried eggs that were red and subspherical, with major and minor axis diameters of 0.5 and 0.4 mm, respectively. One female had 72 eggs on its pleopods.

**Fig 8:**
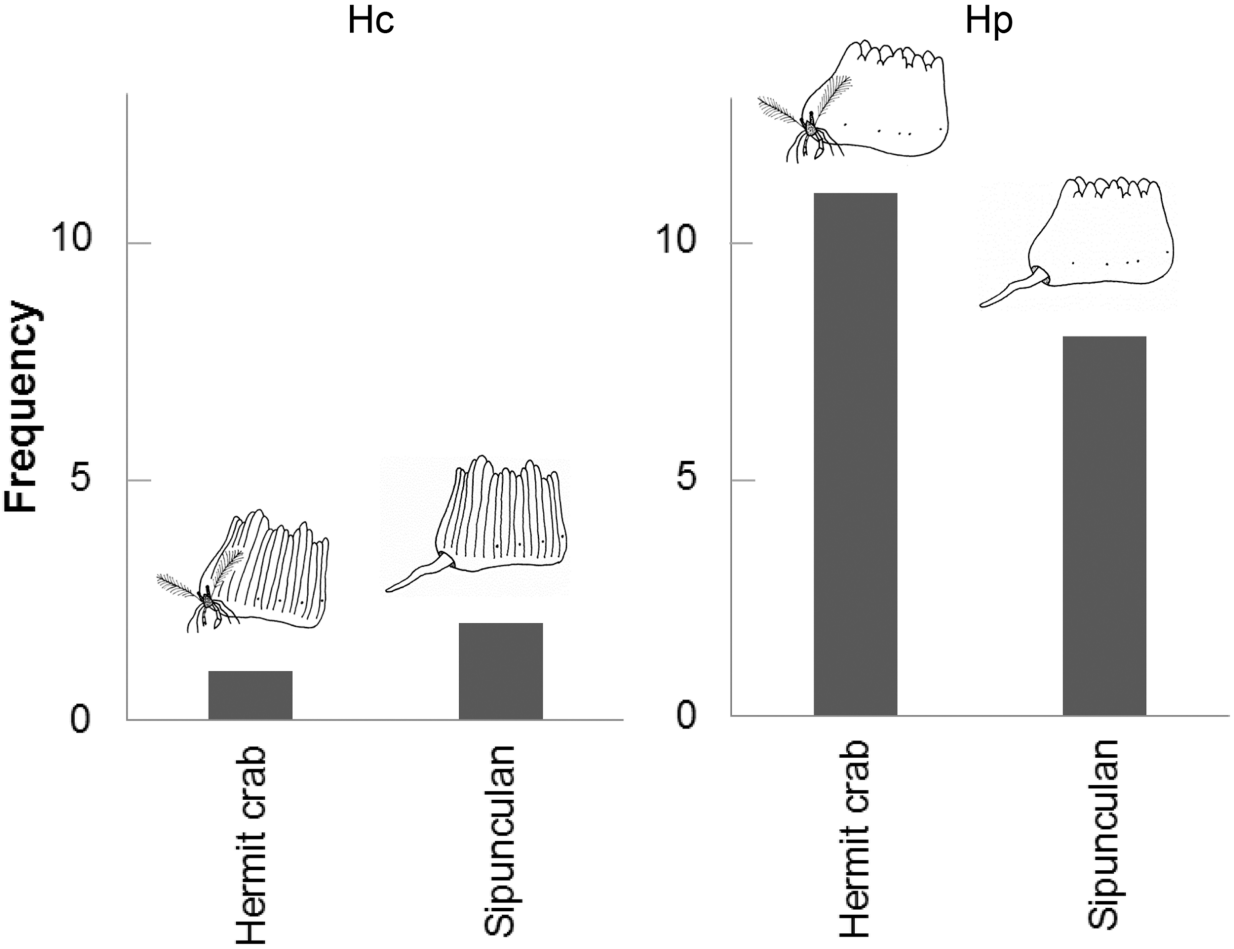
Frequencies of solitary corals symbiotic with a hermit crab/sipunculan in Oshima Strait. Number of solitary corals (Hc, *Heterocyathus alternatus*; Hp, *Heteropsammia cochlea*) inhabited by a hermit crab/sipunculan.

This hermit crab is sympatric with the sipunculan symbiont of walking corals. Among 22 walking corals collected from shallow waters of Oshima Strait between the years 2012 and 2016, 12 (about 55%) were inhabited by hermit crabs, and other 10 by sipunculans (Fig 8). There were no differences in the structures of the corallum cavities between the hermit crab- and the sipunculan-occupied corals. In Oshima Strait, *Heteropsammia cochlea* was thought to be more abundant than *Heterocyathus alternatus* because the former was captured more frequently by dredging.

**Fig 9:**
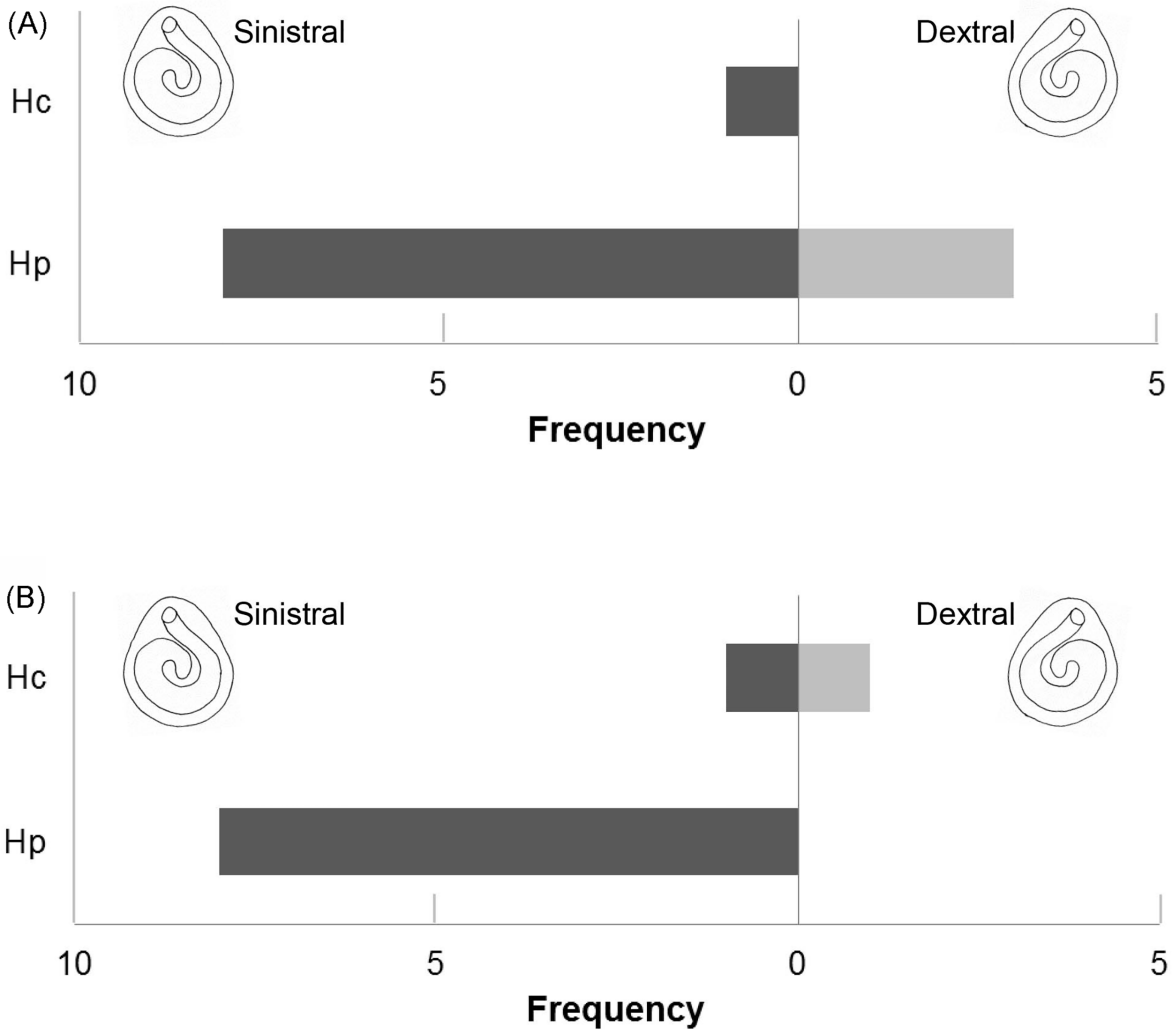
Frequencies of solitary corals having dextrally/sinistrally coiled cavities. Number of solitary corals (Hc, *Heterocyathus alternatus*; Hp, *Heteropsammia cochlea*) having dextrally/sinistrally coiled cavities, in the case of hermit crab-occupied (A) and sipunculan-occupied (B) corals.

Among 12 solitary corals inhabited by the hermit crabs, 3 specimens (25%) had dextrally coiled cavities, and 9 specimens (75%) had sinistrally coiled cavities (Fig 9A). Among 10 solitary corals inhabited by sipunculans, 1 specimen (10%) had a dextrally coiled cavity, and 9 specimens (90%) had sinistrally coiled cavities (Fig 9B). Thus, sinistral coiling was dominant in both hermit crab- and sipunculan-inhabited corals, and there were no significant differences in the rate of sinistrally coiled corals between hermit crab- and sipunculan-inhabited corals (Fisher's exact test, p = 0.368).

## Discussion

In contrast with other congeneric hermit crab species, this new species has a unique lodging that replaces the usual gastropod shell with a living solitary coral that grows with the hermit crab. In the environment, the two studied coral species were also observed to be symbiotic with sipunculans (Fig 8). Because there were no morphological differences between the conspecific corals inhabited by the sipunculan and hermit crab, individuals of both coral species can be symbiotic with either the sipunculan or hermit crab. The fact that the hermit crab is found only in association with the corals but never in naked molluscan shells suggests that the hermit crab is obligately associated with the coral, whereas the coral is not obligately associated with the hermit crab. Among the Indo-Pacific area where these walking corals live, hermit crab-inhabited corals are currently known only from the Amami Islands.

Observations of behavior in aquaria (Fig 7) have shown that the hermit crab transports its symbiotic coral and rescues the coral from being overturned or buried, just as the symbiotic sipunculan does, suggesting that the coral–hermit crab association is the same accommodation–transportation symbiosis observed between the coral and sipunculan. This case is interesting in that an animal species phylogenetically distant from the original symbiont takes over its role transporting the host coral.

Although both the hermit crab and sipunculan play similar roles in transportation and rescue of the host coral, their feeding habits differ. The hermit crab is a suspension or filter feeder, whereas the sipunculan is a detritus feeder found in muddy substrates, suggesting that the microhabitat of each symbiosis may be different.

Furthermore, the question of whether the sipunculan is the original symbiont of the coral in the accommodation–transportation mutualism is intriguing. The complete fit of the sipunculan's body within the corallum cavity [31] and the dominance of sipunculans over the entire distributional range of the corals suggest that the sipunculan is the original symbiont and that the hermit crab is a secondary alternative symbiont. Although little is yet known about the early stages of these symbioses, both the sipunculans and hermit crabs are thought to start their juvenile life by inhabiting vacant minute gastropod shells [15–16].

The extremely slender body of *D*. *heteropsammicola* sp. nov. is considered to be an adaptation to life in the narrow, coiled cavity of the walking coral. The corallum cavity fits the slender body of the symbiotic sipunculan and is narrower and more loosely coiled than that of the gastropod shells utilized by most other hermit crabs. Accordingly, *D*. *heteropsammicola* has likely evolved its slender body to fit the narrow cavity.

The symmetrical telson of this hermit crab is unique among species of *Diogenes*. The symmetry of the telson may be due to the unusual habitat of this species. Because most gastropod shells are coiled dextrally, most gastropod shell-inhabiting hermit crabs have asymmetrical telsons to fit into the dextral shells. On the other hand, the corallum cavity may be coiled dextrally or sinistrally; 25% and 75% of the walking corals inhabited by the hermit crab were dextral and sinistral, respectively (Fig 9). The coexistence of dextral and sinistral corals may be related to the symmetry of the telson.

Why has this saltatory symbiont shift occurred in this accommodation–transportation mutualism? This symbiosis is unique because two different coral species (*Hc*. *aequicostatus* and *Hp*. *cochlea* in Okinawa Island) share two clades of *Aspidosiphon* sipunculans as symbionts [31]. A shift in symbiont acquistion strategy by the corals has allowed the hermit crab to take over the transportation role of the usual sipunculan partners. Our data suggest that the corals are obligately symbiotic with sipunculans and this hermit crab, both of which are also obligately symbiotic with the two genera of the corals. By becoming symbiotic with the corals, the hermit crab has probably gained extra security through protection by coral nematocysts and its more permanent lodging means that it no longer needs to change shells as it grows in size.
